# Assessment of fall-related self-efficacy and activity avoidance in people with Parkinson's disease

**DOI:** 10.1186/1471-2318-10-78

**Published:** 2010-10-25

**Authors:** Maria H Nilsson, Anna-Maria Drake, Peter Hagell

**Affiliations:** 1 Department of Health Sciences, Lund University, Lund, Sweden; 2 Department of Clinical Sciences, Division of Neurosurgery, Lund, Skåne University Hospital, Sweden; 3 Department of Health Sciences, Division of Physiotherapy, Lund University, Lund, Sweden; 4 Department of Neurology, Lund, Skåne University Hospital, Sweden

## Abstract

**Background:**

Fear of falling (FOF) is common in Parkinson's disease (PD), and it is considered a vital aspect of comprehensive balance assessment in PD. FOF can be conceptualized differently. The Falls-Efficacy Scale (FES) assesses fall-related self-efficacy, whereas the Survey of Activities and Fear of Falling in the Elderly (SAFFE) assesses activity avoidance due to the risk of falling. This study aimed at investigating the validity and reliability of FES and SAFFE in people with PD.

**Methods:**

Seventy-nine people with PD (mean age; 64 years, SD 7.2) completed the Swedish version of FES(S), SAFFE and the physical functioning (PF) scale of the 36-Item Short-Form Health Survey (SF-36). FES(S) and SAFFE were administered twice, with an 8.8 (SD 2.3) days interval. Assumptions for summing item scores into total scores were examined and score reliability (Cronbach's alpha and test-retest reliability) were calculated. Construct validity was assessed by examining the pattern of Spearman correlations (r_s_) between the FES(S)/SAFFE and other variables, and by examining differences in FES(S)/SAFFE scores between fallers and non-fallers, genders, and between those reporting FOF and unsteadiness while turning.

**Results:**

For both scales, item mean scores (and standard deviations) were roughly similar and corrected item-total correlations exceeded 0.4. Reliabilities were ≥0.87. FES(S)-scores correlated strongest (r_s_, -0.74, p < 0.001) with SAFFE-scores, whereas SAFFE-scores correlated strongest with PF-scores (r_s_, -0.76, p < 0.001). Both scales correlated weakest with age (r_s _≤ 0.08). Experiencing falls, unsteadiness while turning, and FOF was associated with lower fall-related self-efficacy and higher activity avoidance.

**Conclusions:**

This study provides initial support for the score reliability and validity of the FES(S) and SAFFE in people with PD.

## Background

People with Parkinson's disease (PD) run an increased risk of falling, and most falls occur while walking or turning [1-5]. Some factors associated with an increased risk of falling are longer PD duration, limitations in activities of daily living (ADL) and more severe motor symptoms [2,6-11]. Latt et al. identified abnormal posture, freezing of gait, frontal impairment, impaired balance and reduced knee extensor strength as independent risk factors for future falls [12]. Age does not appear associated with an increased fall risk in people with PD [2,6,8,10,13].

Fear of falling (FOF) and decreased balance confidence is more common in people with PD than in healthy controls [14-16]. In a study investigating 119 people with PD, 59% of the participants reported having FOF [8]. FOF is even more common and pronounced among fallers, and it can cause ADL restrictions and social isolation [8-11,14,17,18]. After adjusting for prior falls and PD-specific impairments, FOF has been shown to be a significant predictor of future falls [19]. It has therefore been recommended to include FOF when assessing balance performance in persons with PD [15,20].

Conceptually, the construct of FOF has been described as an ongoing concern about falling, a loss of balance confidence, a low fall-related efficacy, or as activity avoidance [21-25]. These differences in conceptualization are also reflected in available assessment tools. For example, Tinetti et al. developed the Falls Efficacy Scale (FES) to assess "the degree of perceived efficacy at avoiding a fall during each of 10 relatively nonhazardous activities of daily living" [22]. The Survey of Activities and Fear of Falling in the Elderly (SAFFE), on the other hand asks about activity avoidance due to the risk of falling [24,25].

Although FOF is considered a vital aspect of comprehensive balance assessment in PD, the measurement properties of FES and SAFFE do not appear to have been investigated in PD. This is unfortunate since traditional indices of such properties (e.g. scaling assumptions, reliability and validity) are sample dependent.

A reliable score contains little measurement error and is reproducible. This can be assessed in various ways, of which Cronbachs alpha and test-retest reliability are the most common [26]. Construct validity relates to the extent an instrument produces scores that are representative of the variable it is intended to represent. It includes convergent validity and divergent validity, which can be assessed by examining the pattern of correlations with other variables. In terms of FOF, scores exhibiting relatively strong associations with related aspects (e.g. physical functioning and mobility), but weaker associations with aspects less strongly associated with FOF (e.g. demographic variables) could thus be interpreted in support of convergent and divergent validity, respectively. Construct validity also includes whether scores distinguish between groups that are expected to differ in relation to the investigated construct [27], e.g. fallers and non-fallers.

The aim of this study was to investigate the measurement properties of the self-completed versions of the Swedish FES and SAFFE in people with PD.

## Methods

### Participants

Data were collected from two samples including 79 people with clinically diagnosed idiopathic PD [28] (Table 1). All participants were recruited from a Swedish university hospital, where the patients are regularly evaluated (clinical tests and questionnaires) as either in- or outpatients. Selection of participants was done by the treating neurologist or by a specialist nurse working within the same movement disorder team. Dementia was an exclusion criterion for both samples.

**Table 1:** Participants' characteristics

**Total sample**, n = 79	
Gender (men/women), n (%)	56 (71%)/23 (29%)
Mean age (SD), years	64.5 (7.2)
Mean PD duration (SD), years	15.9 (7.3)
Experienced falls past six months (no/yes), n (%)	27 (34%)/52 (66%)
Fear of falling (no/yes), n (%)	48 (62%)/30 (38%) 1 missing
Unsteady when turning (no/yes), n (%)	26 (34%)/51 (66%) 2 missing
PF, mean (SD)	61.3 (26.2)

**Outpatient clinic sample**, n = 37	

Gender (men/women), n (%)	29 (78%)/8 (22%)
Mean age (SD), years	65.0 (6.3)
Mean PD duration (SD), years	14.3 (8.4)
Experienced falls past six months (no/yes), n (%)	17 (46%)/20 (54%)
Fear of falling (no/yes), n (%)	28 (76%)/9 (24%)
Unsteady when turning (no/yes), n (%)	16 (43%)/21 (57%)
PF, mean (SD)	65.9 (23.6)
UPDRS part II, median (q1-q3)	7.5 (3.0-14.0)
UPDRS part III, median (q1-q3)	15.0 (8.3-21.5)
Timed Up & Go (s), mean (SD)	9.3 (2.6)
Comfortable gait speed (m/s), mean (SD)	1.3 (0.25)
Fast gait speed (m/s), mean (SD)	1.7 (0.36)

**Survey sample**, n = 42	

Gender (men/women), n (%)	27 (64%)/15 (36%)
Mean age (SD), years	64.0 (7.9)
Mean PD duration (SD), years	17.3 (5.8)
Experienced falls past six months (no/yes), n (%)	10 (21%)/32 (68%)
Fear of falling (no/yes), n (%)	20 (50%)/21 (50%) 1 missing
Unsteady when turning (no/yes), n (%)	10(25%)/30 (75%) 2 missing
PF, mean (SD)	57.2 (28.0)

PF: Physical Functioning, which is one of the subscales of SF-36. PF-score can range between 0-100.

UPDRS: Unified Parkinson's Disease Rating Scale. Part II: ADL, where the score can range between 0-52. Part III: motor examination. The total score can range between 0-108 points. Higher UPDRS-scores denote more severe impairments or motor symptoms.

One sample consisted of 50 people with varying degrees of motor complications and/or gait disturbances, receiving out-patient care at two movement disorder neurologists' clinics during one year. Eight patients declined participation and five patients were unable to attend the study visit. The remaining 37 participants responded to the included questionnaires and they were also assessed clinically.

An additional sample consisted of 42 people, who had received and four who were on the waiting list for Deep Brain Stimulation (DBS) in the subthalamic nucleus. They were invited to participate in a postal survey. A total of 93 patients were eligible, but 47 were excluded for the following reasons: age >75 years, dementia, or previous inclusion in the first sample. Forty-six patients were invited to participate in the survey and 42 consented (Table 1).

### Questionnaires

All participants completed two self-administered FOF questionnaires: the Swedish FES, i.e. the FES(S) [29] and SAFFE [25].

Items in the FES(S) are framed "How confident are you that you can....without falling?" [22,23]. The definition of self-efficacy is based on Banduras work, who defines perceived self-efficacy as a "judgment of one's capability to accomplish a certain level of performance" [30]. FES(S) includes 13 activities rated from 0 (not confident at all) to 10 (completely confident), yielding a total score between 0 and 130 (higher scores = better balance confidence in performing the activities) [29]. Compared to the original FES, the FES(S) thus contains three additional items (getting in and out of bed, grooming, and toileting) [29].

SAFFE focuses on undesirable consequences of FOF, i.e. activity restriction [24,25]. The underlying assumption is that fear itself is harmless unless it leads to sedentary behavior or restriction of important activities [24]. SAFFE includes 17 activities with three response options: never avoid, sometimes avoid, and always avoid (scored 1-3, respectively) [25]. Thus, the possible score range is 17-51 and higher scores denote greater avoidance.

In addition, the Physical Functioning (PF) scale of the 36-Item Short-Form Health Survey (SF-36) [31] was used. PF comprises ten physical activity items with an emphasis on mobility. The score can range from 0 to100, and higher scores denote better physical functioning. Participants were also asked whether they had fallen during the past six months (No/Yes), if they were afraid of falling (No/Yes), and if they experienced unsteadiness while turning (No/Yes). When completing the questionnaires, the participants also rated their present mobility as either "good", "good but hyperkinetic" or "bad".

### Procedure

Demographic data was collected through self report (Table 1). Participants then completed the FES(S), SAFFE and PF. When completing FES(S) and SAFFE, they were instructed to make a general estimation when rating.

The participants in the outpatient sample were also assessed clinically. Assessments were scheduled at a time point when they typically felt at their best. Following completion of self-reported questionnaires at time 1, they were assessed with gait tests and the Unified PD Rating Scale (UPDRS). Timed gait tests included the 10 meter walk test (gait speed, m/s) and the Timed Up & Go (TUG) [32]. The ten meter walk test was performed both in comfortable and fast gait speed (starting order randomized). A practice trial was followed by a test trial. TUG was only performed in comfortable gait speed. One practice trial was followed by two test trials (best value analyzed). Assessments also included parts II (Activities of daily living, ADL) and III (motor examination) of the UPDRS [33]. After completing the assessments, participants in the outpatient clinic sample were given an envelope with a second set of the FES(S) and SAFFE to be completed a week later, during a period of good mobility. Each envelope was marked with the applicable date.

Participants in the postal survey were mailed a questionnaire package including the same demographic questions as in the clinic sample, together with the FES(S), SAFFE and PF. A second questionnaire package was sent out three days after their first responses had been received.

The study was approved by the by the Advisory Committee for Research Ethics in Health Education (Lund), and all participants gave their written informed consent.

### Analyses

First, we explored if the assumptions for summing item scores into total FES(S) and SAFFE scores were met [34]. That is, whether item mean scores and standard deviations were roughly similar and if the corrected item-total correlations exceeded 0.3. Whether the items appear to represent a common variable was considered supported if corrected item-total correlations were ≥0.4 [34].

Internal consistency (analyzed by inter-item correlations) and Cronbach's alpha was calculated for scores at time 1 and time 2. Test-retest reliability was analyzed by the intraclass correlation (ICC) coefficient between scores obtained at time 1 and 2. ICC was calculated by using random effect two-way ANOVA with absolute agreement (ICC_2.1_) [35]. Reliability values ≥0.8 are recommended [36]. Any systematic change in mean scores between time 1 and time 2 was scrutinized by ANOVA. In addition, the standard error of measurement (SEM) was estimated using the formula SEM = SD_baseline _× √(1 - ICC).

Floor- and ceiling effects were determined based on participants with computable total scores: The number of participants that score at maximum (or minimum)/the total number of participants, multiplied by 100 [37].

Construct validity of FES(S) and SAFFE was assessed by examining the pattern of Spearman correlations (r_S_) with other variables and differences between subgroups of people. The strongest correlation was anticipated to be between FES(S) and SAFFE since balance confidence, fall-related self-efficacy and activity avoidance all relate to FOF [21-25]. We further anticipated that FES(S) and SAFFE scores would correlate stronger with PF, timed gait tests, UPDRS parts II (ADL) and III (motor symptoms) than with PD-duration and age, of which the latter was anticipated to exhibit the weakest correlation. We expected that FES(S) and SAFFE scores would differ (Mann-Whitney U test) between fallers and non-fallers, genders, and between those reporting FOF and unsteadiness while turning (yes/no). Those affirming the question about previous falls, FOF or being unsteady were anticipated to have a higher degree of FOF than those responding no. In addition, women were anticipated to have a higher degree of FOF than men [38], i.e. higher SAFFE scores and lower FES(S) scores.

All analyses were performed using SPSS 15 (SPSS Inc., Chicago, IL). Two-tailed p-values <0.05 were considered statistically significant. Due to the exploratory nature of the study we did not correct for multiple testing.

## Results

Item and total scores at time 1 and time 2 are presented in Tables 2 and 3. At the first administration, one participant left the FES(S) blank. Mean (SD) FES(S) item scores ranged between 6.2-8.1 (2.6-3.3), and corrected item-total correlations ranged between 0.73-0.89 (Table 2). The average inter-item correlation at time 1 was 0.72 (min-max, 0.46-0.93) and at time 2 it was 0.84 (min-max, 0.69-0.95). Total FES(S) scores could be computed for 88% and 93% of the responding participants at times 1 and 2, respectively. The median (q1-q3) total scores at time 1 and time 2 were 99 (64-121) and 104 (60-126), respectively. Floor and ceiling effects were ≤10.6% at both occasions (Table 2).

**Table 2:** Scores and item-total correlations of the Falls-Efficacy Scale, Swedish version - FES(S), n = 79

		**Time 1, n = 78**^**1**^		**Time 2, n = 71**^**2**^	
**Item**^**a**^	**Activity**	**Mean (SD) n = 78**	**Corrected item-total correlations n = 69**	**Mean (SD)**	**Corrected item-total correlations n = 66**

1	Get in and out of bed	6.7 (3.2) 1 missing	0.82	6.9 (2.8) 1 missing	0.86
2	Get on and off the toilet	7.5 (2.8)	0.82	7.4 (2.7) 1 missing	0.89
3	Personal grooming	8.1 (2.6) 3 missing	0.78	7.8 (2.7) 1 missing	0.90
4	Get in and out of chair	6.8 (3.2)	0.87	7.0 (3.0)	0.96
5	Get dressed and undressed	7.1 (3.0)	0.87	7.2 (3.0)	0.95
6	Take a bath or a shower	7.5 (2.9)	0.85	7.3 (2.9)	0.95
7	Go up and down stairs	7.3 (3.0)	0.82	6.9 (3.0)	0.89
8	Walk around neighborhood	7.1 (3.3) 1 missing	0.73	7.0 (3.3)	0.90
9	Reach into cupboards/closets	6.7 (3.2) 1 missing	0.86	6.8 (3.1) 2 missing	0.91
10	Housecleaning	6.7 (3.1)	0.89	6.8 (3.0)	0.93
11	Prepare simple meals	6.9 (3.0)	0.89	6.6 (3.2)	0.87
12	Answer the telephone	6.2 (3.3) 2 missing	0.87	6.5 (3.3)	0.94
13	Simple shopping	6.2 (3.3) 1 missing	0.81	6.5 (3.4)	0.92

Total score Min-Max		91.6 (34.2) 0-130 n = 69		90.6 (36.4) 13-130 n = 66	
	Skewness (SE)	-0.75 (0.29)		-0.45 (0.30)	
	Kurtosis (SE)	-0.36 (0.57)		-1.22 (0.58)	

^a^How confident/sure are you that you can... *(item wording)*...without falling? Response options range from 0 (not confident at all) to ten (completely confident).

^1^One participant did not respond to FES(S).

^2^Three participants did not complete the FES(S) at time 2. At time 2, data from 5 patients were excluded due to late responses and DBS surgery (see Results).

At time 1, one out of 69 (1.4%) scored 0 and 7 scored 130 (10.1%). At time 2, none of the 66 scored 0 and 7 (10.6%) scored 130.

**Table 3:** Scores and corrected item-total correlations of SAFFE, n = 79

		**Time 1, n = 76 **^**1**^		**Time 2, n = 69 **^**2**^	
**Items**^**a**^	**Activity**	**Mean (SD) n = 76**	**Corrected item-total correlations n = 71**	**Mean (SD) n = 69**	**Corrected item-total correlations n = 62**

1	Go to the shops	1.7 (0.7)	0.81	1.5 (0.7) 2 missing	0.70
2	Clean your house	1.6 (0.6) 1 missing	0.82	1.5 (0.6) 1 missing	0.82
3	Prepare simple meals	1.4 (0.5)	0.69	1.4 (0.5)	0.74
4	Go to the doctor or dentist	1.3 (0.6)	0.69	1.3 (0.5)	0.73
5	Take a bath	1.5 (0.7) 3 missing	0.62	1.5 (0.7) 3 missing	0.74
6	Take a shower	1.3 (0.5)	0.60	1.3 (0.5)	0.72
7	Go for a walk	1.6 (0.7)	0.82	1.6 (0.7)	0.78
8	Go out when it is slippery	2.0 (0.8)	0.76	2.0 (0.7)	0.72
9	Visit a friend or relative	1.4 (0.6) 1 missing	0.83	1.5 (0.7)	0.72
10	Go to a place with crowds	1.8 (0.7)	0.73	1.8 (0.7) 1 missing	0.73
11	Go up and down stairs	1.4 (0.5)	0.69	1.4 (0.5)	0.74
12	Walk around indoors	1.3 (0.5)	0.65	1.3 (0.5) 1 missing	0.69
13	Walk half a mile	1.8 (0.8)	0.76	1.7 (0.8)	0.80
14	Bend down to get something	1.5 (0.6)	0.68	1.6 (0.7) 1 missing	0.69
15	Travel by public transport	1.7 (0.8)	0.77	1.7 (0.8)	0.80
16	Go out to a social event	1.7 (0.6)	0.75	1.6 (0.6)	0.77
17	Reach for something above your head	1.5 (0.6)	0.69	1.6 (0.7)	0.69
	Total score, Mean (SD) Min-max	26.2 (8.4) 17-48 n = 71		25.2 (8.1) 17-46 n = 62	
	Skewness (SE)	0.61 (0.29)		0.80 (0.30)	
	Kurtosis (SE)	-0.67 (0.56)		-0.47 (0.60)	

SAFFE: modified Survey of Activities and Fear of Falling in the Elderly.

^a^For each of the 17 activities, three response options are used: never avoid (coded 1), sometimes avoid (coded 2), and always avoid (coded 3). Thus, the total score can range from 17 to 51 points.

^1^Three participants left the questionnaire blank at time 1.

^2^Three participants did not complete retest (time 2). At time 2, data from 5 patients were excluded due to late responses and DBS surgery (see Results). Two participants left the questionnaire blank.

None reached the maximum score of 51 points. At time 1, 13 out of 71 (18.3%) reached the lowest

possible score (17 points). At time 2, 12 out of 62 (19.4%) attained the lowest possible score.

At the first administration, three participants left SAFFE blank. Mean (SD) SAFFE item scores ranged between 1.3-2.0 (0.5-0.8), and corrected item-total correlations ranged between 0.60-0.83 (Table 3). The average inter-item correlation at time 1 was 0.55 (min-max, 0.27-0.82) and at time 2 it was 0.57 (min-max, 0.34-0.77). Total SAFFE scores could be computed for 93% and 90% of the responding participants at times 1 and 2, respectively. The median (q1-q3) total SAFFE scores at time 1 and time 2 were 24 (18-33) and 24 (18-30). The best possible SAFFE score was attained by 18% of the sample at time 1 and by 19% at time 2; none had the worst possible score (Table 3).

At time 1, 64 out of 79 (81%) participants considered their mobility as "good" or "good but hyperkinetic" when completing the questionnaires. Fourteen rated their mobility as bad. One had missing data.

Score reliabilities and SEM for FES(S) and SAFFE are presented in Table 4. Cronbach's alpha was ≥0.95 for both questionnaires. Seventy-six out of the 79 (96%) participants completed FES(S) and SAFFE twice. However, data from four participants were excluded from the test-retest analyses due to a long delay between times 1 and 2 (≥25 days). An additional participant was excluded due to having undergone DBS-surgery between administrations. For the remaining 71 participants, the mean number of days between test and retest was 8.8 (SD, 2.3). Test-retest reliability (ICC) was 0.87 for FES(S) and 0.92 for SAFFE. None of the scores differed between test occasions (FES(S): F, 0.093; P = 0.762, SAFFE: F, 0.686; P = 0.411). The SEM was 12.3 for the FES(S) and 2.4 for SAFFE.

**Table 4:** Reliability and measurement error of the scores on FES(S) and SAFFE, n = 71

	FES(S)	SAFFE
Cronbach's alpha	0.97/0.99 ^a^	0.95/0.96 ^a^
ICC (95% CI)	0.87 (0.79-0.92)	0.92 (0.86-0.95)
SEM	12.3	2.4

^a ^For time 1/time 2.

ICC: Intraclass Correlation Coefficient (two-way random effects model, absolute agreement).

CI: Confidence interval.

SEM: Standard Error of Measurement; $\text{SD}\cdot \sqrt{1-r}$ (SD: standard deviation of baseline values; r = ICC).

Missing data: Cronbach's alpha can only be calculated for those without any missing item values and where total scores can be computed (see Tables 2 and 3). ICC values were calculated for those having total scores for both test and retest. For FES(S), this applied for 58 participants whereas the corresponding number for SAFFE was 57.

FES(S) scores correlated strongest with SAFFE scores (r_s_, -0.74; p < 0.001). This was followed by significant (p < 0.001) correlations with PF (r_s_, 0.66), fast gait speed (r_s_, 0.63), TUG (r_s_, -0.61), UPDRS parts II (r_s_, -0.58) and III (r_s_, -0.46). Weaker correlations were found between FES(S) and comfortable gait speed (r_s_, 0.30), PD-duration (r_s_, -0.28) and age (r_s_, -0.07).

SAFFE scores correlated strongest with PF (r_s_, -0.76; p < 0.001), followed by significant (p ≤ 0.001) correlations with FES(S) (r_s_, -0.74), TUG (r_s_, 0.67), fast gait speed (r_s_, -0.64), comfortable gait speed (r_s_, -0.52), UPDRS parts II (r_s_, 0.52) and III (r_s_, 0.50). Weaker correlations were attained with PD-duration (r_s_, 0.28) and age (r_s_, 0.08).

Those reporting previous falls, FOF or unsteadiness had significantly (p ≤ 0.017) lower FES(S) scores and higher SAFFE scores than those not reporting these experiences (Table 5). Women scored lower on FES(S) and higher on SAFFE than men (Table 5).

**Table 5:** FES(S) and SAFFE- the effects on gender, falls, fear of falling and unsteadiness

Gender	*Men*	*Women*	*p-value*
FES(S)	108 (82-129), n = 49	83 (52-103), n = 20	0.007
SAFFE	22 (18-29), n = 51	33 (25-38), n = 20	0.001

**Falls**	*Yes*	*No*	

FES(S)	92 (55-120), n = 46	105 (94-129), n = 23	0.017
SAFFE	27 (19-35), n = 47	21 (17-29), n = 24	0.008

**Fear of falling **^1^	*Yes*	*No*	

FES(S)	77 (46-99), n = 27	114 (95-129), n = 41	<0.001
SAFFE	33 (29-38), n = 25	21 (18-26), n = 45	<0.001

**Unsteady turning **^2^	*Yes*	*No*	

FES(S)	83 (54-101), n = 44	120 (108-129), n = 23	<0.001
SAFFE	30 (21-37), n = 44	19 (17-24), n = 25	<0.001

Data are medians and first and third quartiles (q1-q3), and values are only reported for those where total scores could be computed at time 1. At time 1, total FES(S) and SAFFE scores could be computed for 69 and 71 participants, respectively.

^1 ^One missing response.

^2 ^Two missing responses.

FES(S): Falls-Efficacy Scale, Swedish version. The possible score range is 0-130 points, where higher scores denote better balance confidence in performing the activities.

SAFFE: The Survey of Activities and Fear of Falling in the Elderly. The possible score range is 17-51 points and a higher score denote greater avoidance.

## Discussion and Conclusions

This study provides initial support for the scoring assumptions, validity and reliability of the FES(S) and SAFFE among people with PD. This has important clinical implications since FOF is an integral aspect of comprehensive balance assessment in PD.

Our observations provide support for the reliability of SAFFE and FES(S) scores when investigating FOF in people with PD, as alpha and ICC values of both exceeded the recommendations of 0.80 [36]. Cronbach's alpha was ≥ 0.95, and it has been recommended to be at least 0.90 in a clinical application [39]. High alpha values and inter-item correlations may also indicate a redundancy of items [40], suggesting that there may be room for item reduction of the scales, particularly in the FES(S). However, further studies in larger samples addressing this issue more specifically are needed before any firm recommendations can be made. Previous studies investigating the measurement properties of FES(S) and SAFFE have not specifically evaluated people with PD. Hellström and Lindmark investigated the reliability of FES(S) in patients with stroke [29] and found a test-retest reliability of 0.97 (ICC), which is higher than that found here. This may be due to differences in study designs. In the study by Hellstrom and Lindmark the FES(S) was interviewer administrated, whereas it was self-administered in this study. Test-retest reliability for SAFFE has been investigated in the elderly, but over a six month period, with a Spearman correlation coefficient of 0.75 [25]. The time interval (6 months) between test and retest could thus explain the relatively low reliability in that study. This is a particularly relevant aspect since FOF may change over time and may be influenced by external events such as having experienced a fall or a near fall. People with PD have a high risk of falling [1] and about 60 to 75% experience near falls [4,41].

Floor and ceiling effects were below or close to the recommendation of <15-20% [37,42]. This suggests that both scales can be useful in detecting differences over time or between groups. However, measurement errors also need to be taken into consideration as differences in scores need to exceed the measurement error. Several indices have been introduced for the evaluation of measurement errors expressed in absolute values. In this study, we used the SEM which expresses the error of the hypothetical (unknown) "true score" based on the observed raw scores [36]. Although not unchallenged, a number of studies have suggested that the SEM is a reasonable estimate of the minimal important difference [43]. Our FES(S) results suggest that a meaningful change should exceed 12.3 points. The corresponding value for SAFFE was 2.4 points. In order to facilitate interpretation of change scores on an individual level, modern psychometric methods such as Rasch analysis could be used in future studies [44].

The corrected item-total correlations exceeded 0.4 for both FES(S) and SAFFE, which can be taken as support that items of the respective scale represent a common variable [34]. This is an important aspect of validity but it does not tell us what the common variable is. Results from evaluations of construct validity may, however be helpful in this respect. In the present study, the hypothesized patterns of correlations and differences in FOF scores were empirically supported, which provides support for the validity of both FES(S) and SAFFE. However, they do not appear interchangeable. FES(S) correlated the strongest with SAFFE, whereas SAFFE correlated marginally stronger with PF. The correlation coefficient between FES(S) and SAFFE was -0.74. Accordingly, about 55% of the variance in scores on one scale can be explained by scores on the other. This illustrates that although fall-related self-efficacy and activity avoidance are related, they are not interchangeable constructs, and scale selection should be based on the objectives at hand.

FES(S) and SAFFE scores were more strongly correlated with PF-scores and clinical gait tests than to parkinsonian motor symptoms (UPDRS III). This is probably due to the fact that people with PD typically fall while walking [2,3]. Walking has in fact been reported in connection with 54% of all falls in PD [2].

As expected, both FES(S) and SAFFE scores were able to distinguish between those with and without falls, FOF and unsteadiness while turning. Two thirds of those with severe PD experience instability while turning during an everyday task [45]. Turning is furthermore associated with "freezing episodes" (i.e. motor blocks) [46], which are associated with falls [2,7,47]. FOF has previously been reported to be more common and pronounced in people with PD who fall than in non-fallers [8-10,17,18]. Both the FES(S) and SAFFE-scores were able to distinguish between those who reported FOF as well as previous falls. That is, fallers and those reporting FOF had lower fall-related self-efficacy and avoided activities more often due to the risk of falling. Activity avoidance in itself can result in sedentary behaviour which implies the importance of including this construct when evaluating people with PD. Although Koller et al. described that people with PD who fall confined their ambulation [11], this was not evaluated in a systematic way. Bloem et al. described that a restriction of activities due to FOF was more common among people with PD than in controls, but they did not use a standardized questionnaire incorporating a broad variety of activities [14]. To our knowledge, this is the first time activity avoidance in relation to falls has been systematically evaluated in people with PD. The SAFFE items with the highest scores ("more avoidance") were items 8 (go out when it is slippery), 10 (go to a place with crowds), 13 (walk half a mile) and 15 (travel by public transportation). This suggests an impact not only on activity performance but also on participation. The present results furthermore show that people with PD who were unsteady, had experienced previous falls and/or reported a FOF avoided more activities due to the risk of falling. Taken together, these findings indicate the importance of taking this construct into consideration both when evaluating and treating people with PD.

There are some methodological concerns that can affect the external validity of the present findings. For example, demented patients were excluded, and participants had quite a long duration of PD. In addition, the survey sample consisted of selected patients and the clinical sample was not randomly selected. It is also conceivable that responses may be influenced by whether respondents with PD are "on" or "off" while completing questionnaires, which could not be controlled for here since data collection was conducted by postal surveys. However, the high test-retest coefficients argue against this being a major source of bias. Finally, it should be borne in mind that the data presented here was collected through self report and it is unknown whether responses are influenced by mode of administration. The equivalence between self completion and interviewer based administration of the FES(S) and SAFFE needs to be assessed in additional studies.

This study presents initial support for the validity and reliability of the scores of FES(S) and SAFFE in people with PD. Further studies are needed to support or refute the present findings as well as providing firmer evidence for the interpretation of change scores.

## Competing interests

The authors declare that they have no competing interests.

## Authors' contributions

MHN participated in conception and design, acquisition of data, analysis and interpretation of data and drafted the manuscript. A-MH participated in analysis and interpretation of data and in revising the manuscript. PH participated in conception and design, acquisition of data, interpretation of data and in revising the manuscript. All authors read and approved the final manuscript.

## Pre-publication history

The pre-publication history for this paper can be accessed here:

http://www.biomedcentral.com/1471-2318/10/78/prepub
